# Finding the Right Blend of Technologically Enhanced Learning Environments: Randomized Controlled Study of the Effect of Instructional Sequences on Interprofessional Learning

**DOI:** 10.2196/12537

**Published:** 2019-05-28

**Authors:** Sok Ying Liaw, Khoon Kiat Tan, Ling Ting Wu, Seng Chee Tan, Hyekyung Choo, John Yap, Sok Mui Lim, Lilian Wong, Jeanette Ignacio

**Affiliations:** 1 Alice Lee Centre for Nursing Studies National University of Singapore Singapore Singapore; 2 School of Health Sciences Nanyang Polytechnic Singapore Singapore; 3 National Institute of Education, Learning Sciences & Technologies Nanyang Technological University Singapore Singapore; 4 Department of Social Work National University of Singapore Singapore Singapore; 5 National University of Singapore Information Technology National University of Singapore Singapore Singapore; 6 Centre for Learning Environment and Assessment Development Singapore Institute of Technology Singapore Singapore; 7 Department of Pharmacy National University of Singapore Singapore Singapore

**Keywords:** blended learning, constructivism, instructional sequence, interprofessional learning, simulation, technologically-enhanced learning, virtual reality, web-based instruction

## Abstract

**Background:**

With the availability and capabilities of varied technologically enhanced learning activities, the blended learning approach has become increasingly popular in interprofessional education. The combined use of different technologically enhanced learning activities has not been fully examined, particularly to determine the effects of instructional sequences for effective learning outcomes.

**Objective:**

The objective of this study was to investigate whether the instructional sequences of a blended learning approach can improve students’ learning outcomes on interprofessional competencies.

**Methods:**

A randomized controlled study was conducted with 40 interprofessional health care teams. These teams undertook three technologically enhanced learning activities—Web-based instruction (WI), virtual reality (VR), and simulation exercise (SE)—after random assignment to three groups based on three different instructional sequences (WI-VR-SE, WI-SE-VR, and SE-WI-VR). Pretests and posttests were conducted to evaluate the students’ learning outcomes on interprofessional competencies.

**Results:**

A total of 198 participants from the three groups completed the questionnaires. All three groups reported significant improvement in their levels of self-efficacy (*P*<.05) and attitudes (*P*<.001) toward interprofessional team care about 1 month after the interprofessional learning activity. Although no significant difference was found (*P=*.06) between the WI-VR-SE and WI-SE-VR groups in the self-efficacy posttests, participants in the SE-WI-VR group reported significantly lower (*P*<.05) posttest scores than those in the WI-SE-VR group. The majority of the participants (137/198, 69.1%) selected the instructional sequence “WI-VR-SE” as their top preference.

**Conclusions:**

This study shows that the instructional sequence of a blended learning approach can have a significant impact on students’ learning outcomes. The learning of concepts from WI followed by problem-solving activity in the SE was found to be a more effective learning sequence than the reverse sequence. We recommend that future studies focus on scaffolding students’ learning when planning instructional sequences for technologically enhanced learning activities within blended learning environments.

## Introduction

Technologically enhanced learning in health professional education has evolved rapidly from basic text-based learning to inclusion of more multimedia features (eg, video and animation), also known as Web-based multimedia instruction, to provide self-directed learning opportunities for learners on didactic material. Yavner et al [1] described Web-based multimedia instruction as a screen-based set of learning material in a personal computer or other multimedia devices that learners can read, listen to, and watch. Although Web-based multimedia instruction can be used to replace didactic learning methods (eg, lecture), integrating it with group learning activities and experiential learning (eg, patient encounter) can significantly enhance learning [2].

With the development of communication tools, there is a growing trend of electronic learning (e-learning) use in interprofessional learning to support interaction and promote information sharing among different health care professionals [3]. E-learning has been identified as a practical and accessible learning tool to overcome logistical challenges often associated with scheduling interprofessional learning activities across different health care courses [4]. Although the evidence of e-learning in improving interprofessional collaboration is significant, feelings of isolation among learners are commonly reported. As a result of diminishing face-to-face interactions, the use of e-learning may also affect interprofessional interactions/dynamics [5]. Therefore, it will be beneficial to use a blended learning approach that combines e-learning and face-to-face interactions to develop interprofessional competencies. A study by Riesen et al [6] found that a blended learning environment that included online, virtual face-to-face, and traditional face-to-face interactions improved health care students’ interprofessional competencies.

Advanced interactive technologies including game-based learning, virtual patient, and virtual reality have recently gained attention in health care education. A virtual reality environment generated by computers to create 3D realms allows every user to don the role of a virtual avatar and enables him/her to interact with the avatar in a real-time environment [7]. The use of this environment for collaborative learning is gaining popularity in health care education, as more health care professionals are searching for ways to develop their interprofessional collaborative practice competencies, as encouraged by the government and professional bodies. Many were also spurred by the success of the use of physical simulation for team training [8]. Implementing such training in a virtual environment offers several advantages, including overcoming logistical challenges (eg, facilities and scheduling) associated with physical simulation [9]. Studies have shown that the use of virtual simulations was found to be as effective as physical simulation in improving performance in acute care [10,11]. Blending virtual and physical simulations may optimize learning effectiveness [12].

Blended learning refers to combining computer-mediated learning with face-to-face interactions. This can involve a mix of Web-based technologies or various pedagogical approaches to support learning [13]. A systematic review on the effectiveness of blended learning in health professionals showed a consistent positive effect when compared with no intervention, which is better than or at least comparable to nonblended instruction for the acquisition of knowledge [14]. Another systematic review, which focused on the role of blended learning in health care clinical education, reported the potential of learning in improving clinical competencies and suggested that future research should go beyond a mere comparison with traditional approaches [2]. Rather, research into blended learning should pay attention to the ways of implementing a blended course effectively [14], including different blends of effective approaches, tools, and technologies [15].

Although the combined use of different learning modalities within blended learning environments has become increasingly popular in the delivery of interprofessional education [6], it remains unclear whether the instructional sequences of these learning modalities affect learning outcomes. In this study, a technologically enabled blended learning approach was designed to deliver an interprofessional learning activity on patient-centered team care for health care students. Based on the learning processes of concept building, experiential learning, and problem solving, three technologically enabled learning modalities—Web-based instruction (WI), virtual reality (VR) environment, and face-to-face simulation exercise (SE)—were implemented. The conventional instruction sequence often involves scaffolding of students’ learning that starts with the acquisition of content (concept building) using WI, engagement in experiential learning in VR to internalize the learned concepts, and the application of learning to problem solve in SE (ie, WI-VR-SE). Would other alternative sequences such as SE-WI-VR and WI-SE-VR be as effective? An initial exploratory problem-solving SE followed by concept building or experiential learning can unfold benefits by activating learners’ prior knowledge, enhancing their awareness of knowledge gaps, and helping them to relate new knowledge delivered by instructional and experiential learning [16].

This study aimed to investigate whether the instructional sequences of a blended learning approach can improve students’ learning outcomes on interprofessional competencies. The study also aimed to explore students’ evaluations of the different technologically enhanced learning modalities and their instructional sequences for the delivery of interprofessional education.

## Methods

### Study Designs, Setting, and Participants

After obtaining approval from institutional review boards of higher educational institutions, a prospective randomized controlled trial study with a pre-post test design was conducted on students undertaking health care courses (medicine, nursing, pharmacy, physiotherapy, occupational therapy, and medical social work) who were in their senior year at three tertiary educational institutions. Participants were recruited via convenience sampling using email and Facebook. The results of a previous study were used to estimate the sample size [17]. For a moderate effect size, a power analysis suggested at least 52 samples in each group to achieve 80% power at 5% level of significance for one-way analysis of variance testing the differences among the three groups. With an estimate of 20% overall dropout rate, a minimum of 195 students (65 per group) were targeted for recruitment. The recruited participants were assigned to interprofessional teams, each consisting five to six health care students, with one member from each health care course. These interprofessional teams were randomly assigned to one of the three groups: WI-VR-SE (14 teams), WI-SE-VR (13 teams), and SE-WI-VR (13 teams). The grouping allocations were made known to the researcher but concealed from the participants.

### Implementation of Blended Learning Strategies

**Figure 1:**
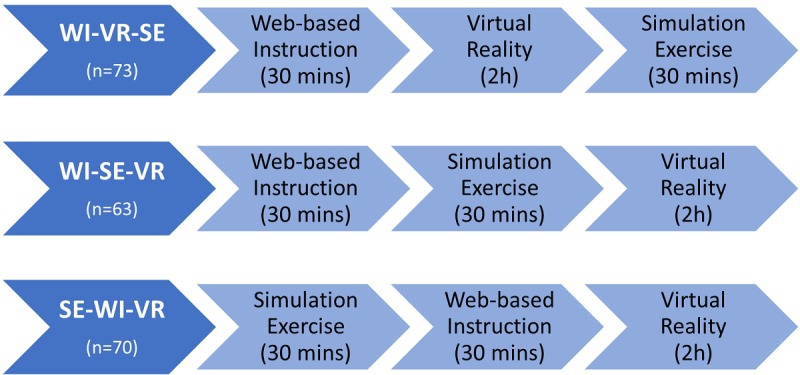
Three blended learning approaches. WI: Web-based instruction; VR: virtual reality; SE: simulation exercise.

The interprofessional training on multidisciplinary rounds for patient-centered team care was selected as the focus of the learning content. As shown in Figure 1, the interprofessional teams were assigned to undertake interprofessional learning using three different instructional sequences (WI-VR-SE, WI-SE-VR, and SE-WI-VR) at a university simulation center. At the WI station, the participants were brought into an individual room with a computer set up. At their own paces, they acquired the concept of team mental models for multidisciplinary rounds by watching a video of a patient case presentation. At the VR and SE stations, the participants formed an interprofessional team of five to six participants to engage in the learning activities in the multidisciplinary rounds. At the VR station, using their own health care professional avatars, the participants worked through virtual simulation scenarios for the delivery of team care on multidisciplinary round. This was followed by debriefing sessions led by a trained facilitator in the VR environment. At the SE station, after spending some time exploring a case study and discussing the plan of care, an interprofessional team would enter a simulated physical ward environment to perform a multidisciplinary round on a simulated patient with physical and psychosocial problems. Taken together, the entire learning process on multidisciplinary rounds for patient-centered team care involved concept building using WI, facilitator-led experiential learning in VR, and the application of learning to problem solve in SE.

### Data Collection and Instruments

The participants were asked to complete questionnaires before and 1 month after the blended learning activity to evaluate their interprofessional competencies. A 5-item confidence scale with a 10-point scale, developed by Grundy [18] for measurement of confidence level related to a performance, was used to measure the participants’ level of self-efficacy in contributing to patient-centered care in an interprofessional team. The Cronbach alpha for this study was 0.79. A 24-item Interprofessional Socialization and Valuing Scale (ISVS) with three subscales (self-perceived ability to work with others, value in working with others, and comfort in working with others) was used to measure the participants’ beliefs, behaviors, and attitudes in interprofessional socialization [19]. Each item was rated on a 5-point Likert scale. A high internal consistency of Cronbach alpha of 0.80 was obtained in this study. A 14-item Attitudes Toward Interprofessional Health Care Teams (ATIHCT) was administered to measure the participants’ attitudes toward health care teams [20]; this study obtained a high internal consistency with a Cronbach alpha of 0.80.

An evaluation questionnaire was administered immediately after the blended learning activity to evaluate three learning modalities in relation to the levels of enthusiasm, help, and satisfaction on a 5-point Likert scale. A question on ranking the three instructional sequences in order of the learner’s preference was included in the evaluation questionnaire. All questionnaires were completed electronically to ensure that participants answered all the questions.

### Data Analysis

Descriptive statistics were used to represent the demographic characteristics of the study population and the evaluation of the learning strategies. Analysis of variance with a posthoc test was carried out to determine the differences between the groups on demographic characteristics. A paired *t* test was used to examine any significant changes between the baseline and posttest attitude scores measured by the ATIHCT and ISVS. Analysis of covariance was employed to evaluate the effect of the blended learning approach on attitudes and self-efficacy posttest scores by using pretest scores as a covariate.

## Results

**Figure 2:**
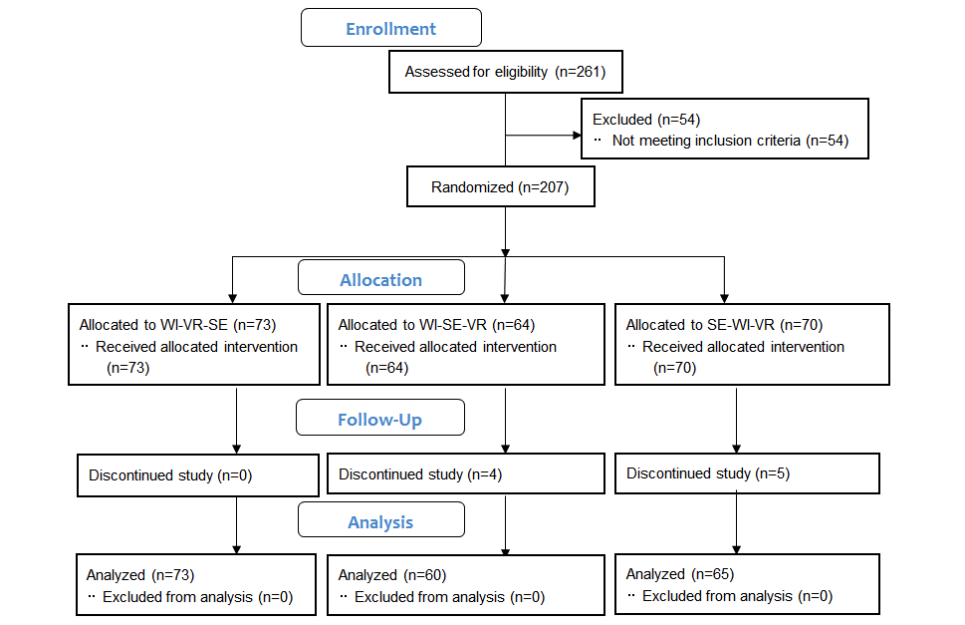
CONSORT diagram. WI: Web-based instruction; VR: virtual reality; SE: simulation exercise.

A total of 207 health care students participated in the blended learning activity. However, only 198 completed the questionnaires (WI-VR-SE: 73; WI-SE-VR: 60, SE-WI-VR: 65), with a response rate of 95.7% for the one-month posttest questionnaires (Figure 2).

As shown in Table 1, most of the participants were female (65.2%) and undertaking a degree course (69.7%). There were no significant differences in demographic characteristics among the three groups, including age (*P*=.62), gender (*P*=.81), type of qualification (*P*=.81), and type of health care course (*P*=.21). This suggested homogeneity of the participants between the three groups.

**Figure 3:**
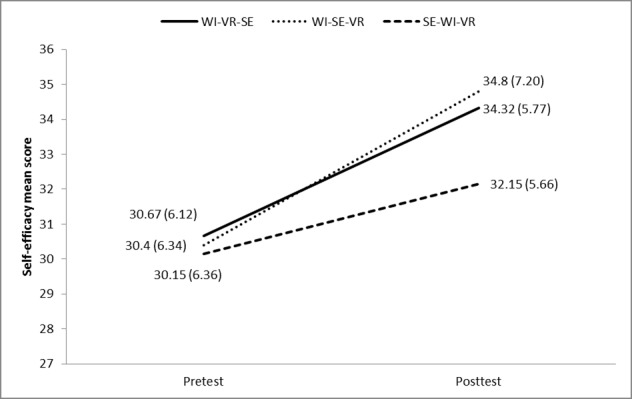
Mean (SD) self-efficacy scores at pretest and posttest. WI: Web-based instruction; VR: virtual reality; SE: simulation exercise.

As shown in Figure 3, all three groups reported significantly higher levels (*P*<.05) of self-efficacy in performing interprofessional team care after interprofessional learning. However, the SE-WI-VR group had the lowest self-efficacy posttest mean scores. Between-group comparisons using analysis of covariance revealed a significant difference (*P*=.03) among the three groups in terms of self-efficacy posttest mean scores, with the SE-WI-VR group reporting significantly lower posttest scores than the WI-SE-VR groups after controlling the pretest scores (*P*=.04). There were no significant differences between the WI-VR-SE and WI-SE-VR groups in self-efficacy posttest scores after controlling the pretest scores (*P*=.06).

Table 2 shows that the posttest scores on interprofessional socialization using the ISVS increased significantly (*P*<.001) from the baseline scores for all three groups. However, no significant differences were found between the baseline and posttest scores on the ATIHCT for all three groups. Between-group comparisons also did not identify any significant differences in the attitude posttest scores for both the ISVS and ATIHCT after controlling the pretest scores.

As shown in Table 3, the mean score ratings on 5-point scales indicated that the participants were enthusiastic, satisfied, and able to perceive the helpfulness of the individual learning strategies. Among them, SE had the highest mean scores for level of help (mean 4.19, SD 0.79), level of satisfaction (mean 4.17, SD 0.79), and level of enthusiasm (mean 3.99, SD 0.68).

**Table 1:** Demographic characteristics.

Characteristic		Overall	WI^a^-VR^b^-SE^c^ group (n=73)	WI-SE-VR group (n=60)	SE-WI-VR group (n=65)	*P* value
Age, mean (SD)		22.60 (1.73)	22.73 (1.83)	2263 (2.08)	22.44 (1.20)	0.62
**Gender, n (%)**						**0.81**
	Male	69 (34.8)	27 (37.0)	19 (31.7)	23 (35.4)	
	Female	129 (65.2)	46 (63.0)	41 (68.3)	42 (64.6)	
**Type of qualification, n (%)**						**0.81**
	Degree	138 (69.7)	49 (67.1)	47 (78.3)	42 (64.6)	
	Diploma	60 (30.3)	24 (32.9)	13 (21.7)	23 (35.4)	
**Type of health care course, n (%)**						**0.21**
	Medicine	38 (19.2)	14 (19.2)	13 (21.7)	11 (16.9)	
	Nursing	36 (18.2)	14 (19.2)	10 (16.7)	12 (18.5)	
	Pharmacy	38 (19.2)	14 (19.2)	12 (20.0)	12 (18.5)	
	Occupational therapy	29 (14.6)	11 (15.1)	9 (15.0)	9 (13.8)	
	Physiotherapy	22 (11.1)	7 (9.6)	6 (10.0)	9 (13.8)	
	Social work	35 (17.7)	13 (17.8)	10 (16.7)	12 (18.5)	

^a^WI: Web-based instruction.

^b^VR: virtual reality.

^c^SE: simulation exercise.

**Table 2:** Pretest and posttest scores on Attitudes Toward Interprofessional Health Care Teams and Interprofessional Socialization and Valuing Scale.

Instrument (range of possible scores) and group		Baseline, mean (SD)	Post score, mean (SD)	Change of scores (post-pre score), mean (SD)	Within group difference, *t* (*df*)	Between group, *F* (*df*)
**Interprofessional Socialization and Valuing Scale (24-168)**						**0.22 (2)**
	WI-VR-SE	124.49 (14.10)	137.25 (16.87)	12.75 (14.49)	7.52 (72)^a^
	WI-SE-VR	123.92 (14.99)	135.77 (20.05)	11.85 (20.41)	4.49 (59)^a^
	SE-WI-VR	121.94 (13.57)	137.65 (12.41)	15.70 (16.55)	7.65 (64)^a^
**Attitudes Toward Interprofessional Health Care Teams (14-70)**						**0.33 (2)**
	WI-VR-SE	55.26 (5.38)	56.44 (6.94)	1.18 (6.43)	1.57 (72)
	WI-SE-VR	56.02 (5.12)	56.45 (6.31)	0.43 (5.59)	0.60 (59)
	SE-WI-VR	55.42 (5.66)	55.60 (7.14)	0.18 (6.23)	0.24 (64)

^a^*P*<.001

**Table 3:** Participants’ evaluation of the learning strategies.

Evaluation items and learning strategies		Mean score (SD)
**Level of enthusiasm**		
	Simulation exercise	3.99 (0.68)
	Virtual reality	3.92 (0.63)
	Web-based instruction	3.61 (0.68)
**Level of help**		
	Simulation exercise	4.19 (0.79)
	Virtual reality	4.07 (0.73)
	Web-based instruction	3.75 (0.84)
**Level of satisfaction**		
	Simulation exercise	4.17 (0.78)
	Virtual reality	4.03 (0.73)
	Web-based instruction	3.90 (0.73)

In terms of the participants’ top preferences for interprofessional learning through the three different instructional sequences of a blended learning approach, the majority of the participants (69.1%) selected the instructional sequence “WI-VR-SE” as their top preference; in addition, less than 10% of the participants chose the sequence “SE-WI-VR” as their top preferences and about 20% chose the sequence “WI-SE-VR.”

## Discussion

The evaluation of the participants’ levels of self-efficacy in contributing to interprofessional patient-centered care, which was conducted 1 month following the interprofessional learning activity, indicated significant improvements from the baseline scores for all three groups. Drawing from the constructivist learning theory, case-based and problem-based learning approaches were incorporated into the blended learning strategies to help learners develop competencies in performing interprofessional patient-centered care. These approaches are known to enable participants to actively construct their knowledge based on their interpretations of experiences [21]. Furthermore, according to the situated learning theory, which is another aspect of constructivism [22], situating learning in authentic contexts that reflect the way knowledge and skills can be applied in actual life provides learners with meaningful learning experiences and thus deepens their learning [23]. As SE provided the most authentic learning environment, participants reported that it contributed to the highest levels of help and satisfaction toward their learning.

Among the three groups, participants who undertook the SE-WI-VR sequence were found to have the lowest improvement of self-efficacy levels, which was significantly lower than those who undertook WI-SE-VR. These findings suggest that the instructional sequences of a blended learning approach can significantly influence learners’ perceptions of learning outcomes. Our findings show that learning starting with instructional prompts on concepts followed by problem solving in SE was a more effective learning sequence than the reverse sequence. Although the problem-solving activity in SE may prepare learners to benefit from WI learning, the introduction of concepts seemed to be necessary, particularly for novice health care students who may not have sufficient domain knowledge to engage in problem-solving tasks in SE [16]. This study therefore supports the introduction of concepts on team mental models, as they serve as cognitive tools that scaffold students’ abilities to perform tasks on interprofessional team care delivery [24].

Although no significant differences in self-efficacy scores were found between the instructional sequences WI-SE-VR and WI-VR-SE, the instructional sequence WI-VR-SE was chosen by the majority of the students as their top preferences. In this WI-VR-SE instructional sequence, scaffolding support was provided initially in WI through a video demonstration of desired performances using cognitive tools. This was followed by facilitator-led experiential learning in VR that fostered reflection and metacognition. The support was gradually decreased in SE, where students were given the opportunity to collaborate among themselves to apply their learning to problem solve a case scenario. According to Kim and Hannafin [25], in the context of technologically enhanced learning, scaffolding is defined as “cognitive and social supports designed to augment student problem-solving inquiry.” Our findings therefore support a previous study on the use of scaffolding to facilitate the alignment of technologically enhanced learning activities in blended learning [25].

Unlike self-efficacy in performing interprofessional team care, no significant differences were reported among the three groups in their attitudes toward interprofessional socialization and the health care team. Given the opportunities to engage in collaborative learning in VR and SE, all groups reported a significant improvement in attitudes toward interprofessional socialization. According to the social constructivist views of learning, the exchange and discussion of ideas through social interaction are critical for learners to construct meaningful knowledge [26]. Although SE provided a more authentic social learning environment, most students in our study preferred to undertake interprofessional learning in VR before engaging in SE. A possible reason could be that, unlike face-to-face social interactions, the anonymity embedded in VR may provide a less stressful and less threatening learning environment for different health care students to communicate and collaborate with one another. The presence of stress in simulation training was commonly reported in previous studies [27]. The study therefore supports the use of VR to prepare students for physical simulation.

Although a robust study using a randomized controlled trial and a large sample size was employed in this study, an important limitation is the lack of performance measurement as the outcome measure. The use of self-reported surveys that measure changes in attitudes and self-perceived efficacy may not predict actual performances and may be subjected to social desirability. Future studies can measure the impact on team performance. Another limitation is that we did not incorporate a debriefing or feedback session in the SE after the students’ role-play experiences, which could have served as expert feedback and engage learners in reflection on actions.

In conclusion, this study provides evidence of the effectiveness of a blended learning approach using a randomized controlled study. Technologically enhanced learning strategies based on the constructivist learning theory improves health care students’ interprofessional competencies. The study suggests that the instructional sequence of a blended learning approach can have a significant impact on students’ learning outcomes. The more effective learning sequence allows learners to grasp concepts from WI before presenting them with a problem-solving activity in SE. From the learners’ perspective, they preferred to start with concept building using WI, followed by experiential learning in VR and subsequent application through SE. This study provides recommendation for future practice to scaffold students’ learning when planning the instructional sequence of technologically enhanced learning activities within blended learning environments. Future studies can undertake a more robust outcome measurement by evaluating the effects of the instructional sequence on team performance.

## Abbreviations

ATIHCT: Attitudes Toward Interprofessional Health Care Teams
ISVS: Interprofessional Socialization and Valuing Scale
SE: simulation exercise
VR: virtual reality
WI: Web-based instruction

## Appendix

Multimedia Appendix 1 CONSORT-EHEALTH checklist (V 1.6.1).
